# Water Soluble Iron-Based Coordination Trimers as Synergistic Adjuvants for Pancreatic Cancer

**DOI:** 10.3390/antiox10010066

**Published:** 2021-01-07

**Authors:** Marco Cordani, Esther Resines-Urien, Arturo Gamonal, Paula Milán-Rois, Lionel Salmon, Azzedine Bousseksou, Jose Sanchez Costa, Álvaro Somoza

**Affiliations:** 1 IMDEA Nanociencia, Faraday 9, Ciudad Universitaria de Cantoblanco, 28049 Madrid, Spain; marco.cordani@imdea.org (M.C.); esther.resines@imdea.org (E.R.-U.); arturo.gamonal@imdea.org (A.G.); paula.milan@imdea.org (P.M.-R.); 2 Laboratoire de Chimie de Coordination, UPR8241, 205 Route de Narbonne, CEDEX 4, 31077 Toulouse, France; lionel.salmon@lcc-toulouse.fr (L.S.); azzedine.bousseksou@lcc-toulouse.fr (A.B.)

**Keywords:** pancreatic cancer, antitumor agents, coordination polymers, bioinorganic chemistry, reactive oxygen species

## Abstract

Pancreatic cancer is a usually fatal disease that needs innovative therapeutic approaches since the current treatments are poorly effective. In this study, based on cell lines, triazole-based coordination trimers made with soluble Fe(II) in an aqueous media were explored for the first time as adjuvant agents for the treatment of this condition. These coordination complexes were effective at relatively high concentrations and led to an increase in reactive oxygen species (ROS) in two pancreatic cancer cell lines, PANC-1 and BXPC-3, and this effect was accompanied by a significant reduction in cell viability in the presence of gemcitabine (GEM). Importantly, the tested compounds enhanced the effect of GEM, an approved drug for pancreatic cancer, through apoptosis induction and downregulation of the mTOR pathway. Although further evaluation in animal-based models of pancreatic cancer is needed, these results open novel avenues for exploring these iron-based materials in biomedicine in general and in pancreatic cancer treatment.

## 1. Introduction

Pancreatic ductal adenocarcinoma (PDAC) is an orphan disease with a terrible prognosis, even when diagnosed early. The survival rate after five years remains below 5%, despite tremendous efforts at the preclinical and clinical stages. Worldwide, in 2018 there were 460,000 new cases of pancreatic cancer and 430,000 associated deaths [1]. The early symptoms of the disease are extremely rare, and, therefore, most patients present with locally advanced disease and/or metastasis at the time of diagnosis, limiting their options of being treated by surgery [2]. The standard treatment for the advanced disease includes gemcitabine (2′,2′-difluoro-2′-deoxycytidine; GEM), with a response rate of less than 20% [3]. Therefore, the identification of novel therapeutic strategies to overcome the limitations of current therapies against PDAC is urgent [4].

In this regard, we are exploring the use of coordination polymers (CPs) as adjuvants for the treatment of pancreatic cancer. These materials are built from metal ions linked by ligands, forming different topologies ranging from 1D to 3D structures. A great variety of CPs have been prepared with different properties and functionalities, such as porous frameworks for gas sorption and separation, catalysis, molecular and ion-exchange, molecular magnets, luminescence, among others [5]. The biological applications of these materials have been limited mainly due to their low solubility in biological media. Nevertheless, they are extremely appealing due to the versatility that confers the use of different biocompatible metals or ligands [6]. Thus, CPs have been employed as nanocarriers [7,8,9,10,11], to control the release of therapeutics [12,13,14,15], as contrast agents in MRIs [16,17] or optical imaging [18], and even in theranostic approaches [6].

A particularly interesting family of iron CPs is the chain-like polymeric Fe(II) system, with general formula {[Fe(Rtrz)3]X2}n, (Rtrz = substituted triazole; X = monovalent anion), which have been the focus of remarkable studies in a solid state [19]. These Fe(II) systems present the physical phenomenon of spin-crossover, which is accompanied by a strong thermochromism and a magnetic transition between diamagnetic and paramagnetic behavior in the solid state either as powder [20,21] or as nanoparticles [22]. However, less attention has been paid to the phenomenon in an aqueous solution [23], mainly due to their poor solubility [24]. To overcome this limitation, shorter polymeric chains (more precisely, trinuclear oligomers) are used in this report, providing water-soluble derivatives.

Reactive oxygen species (ROS) are highly reactive molecules containing oxygen produced by cellular metabolism, and they participate in several biological processes, including signal transduction, enzyme activation, gene expression, and protein post-translational modifications [25]. However, although the role of ROS in cellular physiology is well established, when produced in excess, they may cause irreversible cellular damage through the oxidation of biomolecules such as lipid membranes, enzymes, or DNA, which generally leads to cellular death [26]. ROS can also lead to carcinogenesis and tumor progression by inducing DNA mutations, genomic instability, and pro-oncogenic signaling pathways [27]. In this regard, cancer cells frequently exhibit increased ROS, mainly resulting from their high metabolic activity and oncogene activation [27]. However, this increased oxidative stress can promote cell proliferation without leading to cell death, but may make cancer cells vulnerable to exogenous oxidizing agents that generate additional ROS, which may increase oxidative stress levels above the cytotoxic threshold [28]. For this reason, the overstimulation of ROS by anticancer agents has been largely exploited and represents a main mechanism through which chemotherapy drugs kill cancer cells [29,30].

In this regard, once the oligomers were synthesized and characterized, their effect on cell viability and ROS induction, in the presence or absence of GEM, were assessed in two pancreatic cancer cell lines (PANC-1 and BXPC-3) as well as in a non-tumoral cell line (HaCaT). Furthermore, the oligomer’s (or CP’s) effect on apoptosis and the mTOR pathway within pancreatic cancer cells was evaluated to dissect the molecular and functional aspects underlying the mechanism of action of such structures. Finally, the involvement of ROS in the regulation of cell viability and the mTOR pathway was analyzed. Overall, this study provides new insight into the molecular mechanisms of action of CPs, which could be exploited as adjuvants for the treatment of pancreatic cancer.

## 2. Materials and Methods

### 2.1. Chemicals

Roswell Park Memorial Institute (RPMI) medium, Dulbecco’s Modified Eagle’s (DMEM) medium, streptomycin–penicillin (100×), fetal bovine serum (FBS), l-glutamine (100×), trypsin (10×), phosphate-buffered saline (PBS), and cell culture plasticware were purchased from VWR. Gemcitabine was purchased from Fluorochem. *N*-acetyl cysteine was purchased from Sigma Aldrich, Saint Louis, MO, USA. Chemicals and reagents were purchased from commercial suppliers and used as received following the indications reported.

### 2.2. Cell Lines and Culture Conditions

PANC-1 and HaCaT cells were purchased from American Type Culture Collection (ATCC, Rockville, MD, USA) and cultured in DMEM medium with 10% FBS, 1% streptomycin–penicillin, and 1% l-glutamine at 37 °C in a Binder CB210 incubator (5% CO_2_). BXPC-3 was a gift from Ibane Abasolo Olaortua (CIBBIM-Nanomedicine Institut de Recerca Hospital Universitari Vall d’Hebron) and was cultured in RPMI medium with 10% FBS, 1% streptomycin–penicillin, and 1% l-glutamine at 37 °C in a Binder CB210 incubator (5% CO_2_). All the procedures were performed inside a laminar flow hood Telstar CV-30/70 (Telstar, Terrassa, Spain).

### 2.3. Analysis of ROS

The non-fluorescent diacetylated 2′,7′-dichlorofluorescein (DCF-DA) probe (Sigma Aldrich, Saint Louis, MO, USA), which becomes highly fluorescent upon oxidation, was used to evaluate ROS production.

The ability of trimers to generate ROS in free-cell water was assessed after their incubation at three different concentrations (1 μM, 100 nM, or 1 nM) with 1 µM DCF-DA for 48 h. Salt and hydrogen peroxide were incubated with the probe as control. DCF-DA fluorescence was measured by using a multimode plate reader (λexc = 485 nm and λem = 535 nm) (Synergy H4 Hybrid reader (BioTEK, Winooski, VT, USA).

To evaluate ROS activity of C1, C2, and C3 in cellular models, the cell lines were plated in 96-well plates (5 × 10^3^ cells/well) and 24 h later were treated with various compounds and with 5 μM DCF-DA for 4 min at 37 °C. After incubation, cells were washed with 1× PBS (phosphate buffered saline), pH 7.4 (VWR), and DCF-DA fluorescence was measured by using a multimode plate reader (λexc = 485 nm and λem = 535 nm) (Synergy H4 Hybrid reader (BioTEK)). Values were normalized on cell proliferation by an alamarBlue viability assay. Representative images of cells were obtained using a Leica DMI3000 M inverted microscope (Leica, Wetzlar, Germany) at 20× magnification. Images were analyzed using ImageJ software (NIH Image, Bethesda, MD, USA).

### 2.4. Alamar Blue Viability Assay

Cells were seeded in 96-well plates and the day after were incubated with various coordination compounds at the indicated conditions (see figure legends). At the end of the treatments, a stock solution of resazurin sodium salt (Sigma-Aldrich, St. Louis, MO, USA) (1 mg/mL) in PBS was diluted 1% (*v*/*v*) in complete DMEM or RPMI medium and added to the cells. After 3 h in the incubator (37 °C), the fluorescence was measured at 25 °C in a plate reader Synergy H4 Hybrid reader (BioTEK), λex = 550 nm, λem = 590 nm.

The fluorescent intensity measurements were processed using the following Equation:

% Cell viability = ((Sample data − Negative control)/(Positive control − Negative control)) × 100

The positive control corresponded with untreated cells. A resazurin solution without cells was used as a negative control.

### 2.5. Western Blot Analysis

Cells were harvested, washed in PBS, and re-suspended in RIPA buffer (Tebu-BIO #AR0105) in the presence of a protease inhibitor cocktail (Thermo Scientific™, Madrid, Spain #A32955). After incubation on ice for 30 min, the lysates were centrifuged at 14,000× *g* for 10 min at 4 °C and the supernatant fractions were used for Western blot analysis. Protein concentration was measured by Bradford reagent (Bio-Rad protein assay) using bovine serum albumin as a standard. Protein extracts (30 μg/lane) were resolved on a 10% SDS-polyacrylamide gel and electro-blotted onto PVDF membranes (AmershamTM ProtranTM 0.45 μm NC). Membranes were blocked in 5% low-fat milk in TBST or 5% BSA (50 mM Tris pH 7.5, 0.9% NaCl, 0.1% Tween 20) for 1 h at room temperature and probed overnight at 4 °C with a rabbit polyclonal anti-Bcl-2 (1:1000) (Cell Signaling, #2872), rabbit polyclonal anti-p70 S6 kinase (1:1000) (Cell Signaling, #9202), rabbit polyclonal anti-phospho-p70 S6 kinase (Ser371) (1:1000) (Cell Signaling, #9208), or mouse monoclonal anti-GAPDH (1:1000) (Santa Cruz, sc-47724). Horseradish peroxidase conjugated anti-mouse or anti-rabbit IgGs (1:5000 in blocking solution) (Santa Cruz, Spain) were used as secondary antibodies. Immunodetection was carried out using Bio-Rad chemiluminescent substrates and recorded using a Gel Documentation System (Syngene).

### 2.6. Acquisition of NMR Spectra

NMR spectra were recorded on a Bruker Advance 300 (^1^H: 400 MHz) spectrometer at 298 K using partially deuterated solvents as internal standards. Chemical shifts (δ) are denoted in ppm. Multiplicities are denoted as follows: s = singlet, d = doublet, t = triplet.

### 2.7. Acquisition of FT-IR Spectra

FT-IT spectra were recorded as neat samples in the range 400–4000 cm^−1^ on a Bruker Tensor 27 (ATR device) Spectrometer.

### 2.8. Powder X-ray Diffraction Collection

PXRD data were collected in a Rigaku Smartlab SE diffractometer with a Bragg-Brentano configuration, using Cu-Kα radiation (λ = 0.1541 nm). Samples were measured between 5° and 50° with a speed of 1.8° min^−1^ under an X-ray fluorescence reduction mode, at room temperature.

### 2.9. Analysis of Synergy/Antagonism from Combination Studies

To determine possible additive and synergistic effects when using combinations of **C-1**, **C-2** and **C-3** with GEM, the data from cell viability assays were first analyzed using the freely available software Combenefit [31], which simultaneously assesses synergy/antagonism using three published models (Highest single agent (HSA), Bliss, and Loewe).

Three concentrations were employed for **C-1**, **C-2**, and **C-3** (25, 500, and 1000 µM), while six concentrations were employed for GEM (0.5, 4.5, 20, 40, 60, 80 µM). All data were normalized to untreated controls, and imported into Combenefit software, where the Loewe Additivity model was employed to identify synergistic, additive, or antagonistic drug combinations. The software calculates the combination index (CI) for each drug combination, where a CI value < 1 indicates synergy, CI = 1 is additive and CI > 1 indicates antagonism.

### 2.10. Annexin-V Assay

Cells were seeded in 6-well plates and the day after were incubated with various coordination compounds at the indicated conditions (see figure legends). At the end of the treatments, the dead cells were collected and the attached ones were trypsinized and collected. The cells were washed with PBS 1× in suspension by centrifugation at 177× *g* for 5 min. Cells were resuspended in 100 µL binding buffer 1× and then 10 µL annexin V 1:10 was added and the cells were incubated for 15 min at 4 °C in darkness. After that, 380 µL binding buffer 1× were added to the samples and then 10 µL propidium iodide. The acquisition was performed in a Beckman Coulter Cytomics 500 Flow Cytometer using 20,000 cells in the Flow Cytometry Service at the CNB-CSIC.

### 2.11. Statistical Analysis

Comparisons among groups were analyzed via the independent-samples one-factor ANOVA test using Prism GraphPad software. All statistical data were obtained using a two-tailed student’s *t*-test and homogeneity of variance tests (*p* values < 0.05 were considered significant).

## 3. Results

### 3.1. Synthesis and Characterization of Three Iron-Based Trimers

**Figure 1:**
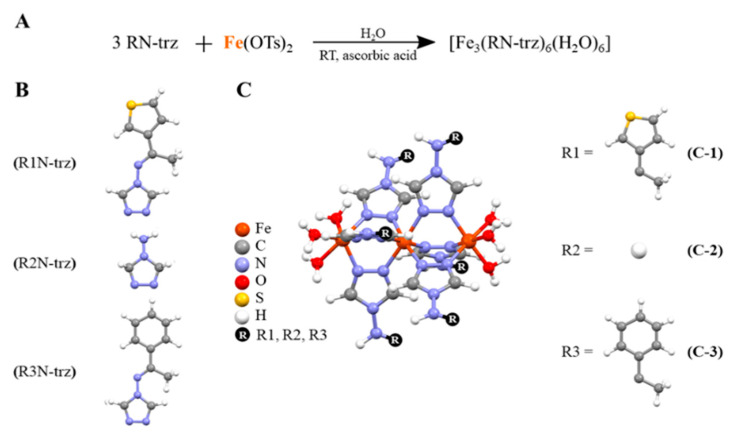
(**A**). Reaction required to obtain the trimers. (**B**). Representation of the different triazole derivatives employed (**C**). Schematic representation of the iron triazole-based trimers, where Fe is represented in orange, C in grey, N in blue, O in red, S in yellow, H in white, and the R group in black.

As mentioned previously, an elegant alternative to employ insoluble CPs in biological media is the use of the triazole-based trimer system [Fe_3_(NH_2_-trz)_6_]X_6_ and its derivatives [Fe_3_(RN-trz)_6_]X_6_. These systems are characterized by a backbone of three linearly arranged Fe(II) ions connected by three triazole ligands (Figure 1) [32,33,34]. The reduced length contributes to their solubility in contrast to that of the larger CPs. In addition, the steric hindrance of the ligands and the strong Fe-*N* coordination bond contribute to the stabilization of the bivalent iron under physiological conditions. The synthetic approach to obtain these water-soluble trinuclear systems resides in the use of p-toluenesulfonate (OTs) as counterions in highly diluted conditions to prevent or limit the formation of larger “Fe(II)/1,2,4-triazole” polymeric chains.

Three different Fe(II) trinuclear triazole-based CPs were synthesized in air conditions using ascorbic acid as the antioxidant, with general structure [Fe_3_(RN-trz)_6_(H_2_O)_6_], using iron(II) tosylate salt and triazole-based ligands: [(E)-*N*-(1-(thiophen-2-yl)ethylidene)-4H-1,2,4-triazol-4-amine (**C-1**), 4-amino-1,2,4-triazole (**C-2**), and (E)-*N*-(1-phenylethylidene)-4H-1,2,4-triazol-4-amine] (**C-3**) (see Figures S1–S5). The stability of these in situ generated complexes in water and phosphate-buffered saline (PBS) was monitored through ESI-MS and specifically with UV-Vis spectra at the incubation conditions (i.e., at 10^−5^ mol·L^−1^, 37 °C and the spectra were taken at 0 h, 24 h, and 48 h) (Figures S6 and S7). It is noteworthy that the enhancement of the intensity of the band around 250 nm takes place without any shift, and also there is an absence of additional bands, which ratifies the stability of the CPs under these conditions. To further characterized these CPs, the solution was evaporated until precipitation and the resulting powders were characterized by FTIR and PXRD (See Figures S8–S11).

### 3.2. Antitumoral Activity of Coordination Polymers

**Figure 2:**
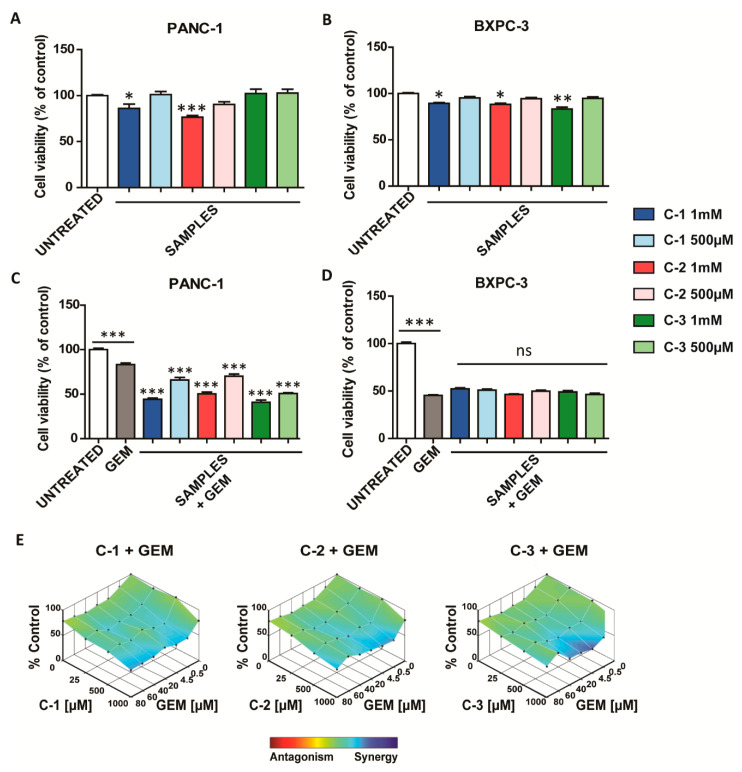
Antitumoral activity of coordination polymers (CPs). (**A**,**B**) Cell viability studies in pancreatic cancer cell lines treated with **C-1, C-2**, and **C-3** for 48 h. (**C**,**D**) Cell viability studies in pancreatic cancer cell lines treated with 4.5 µM gemcitabine (GEM) and **C-1**, **C-2**, and **C-3** for 48 h. The values of treated cells were normalized to the untreated controls and reported as mean ± SEM. Statistical analysis was performed using one-way ANO VA (each group vs. control). (*** *p* < 0.001, ** *p* < 0.01, * *p* < 0.05). (**E**) Combenefit-mapped surface output for the drug combinations involving **C-1**, **C-2**, and **C-3** with GEM in PANC-1 cells. The concentrations of each drug are plotted along the horizontal axes, while the percentages of cells remaining relative to untreated controls are plotted on the vertical axes. A heat map is used to represent the level of synergy (blue color) at each concentration. A heat map is used to represent the level of synergy (blue color) at each concentration. All experiments were conducted at least three times.

Once the trimers were obtained, their effect on cell viability was evaluated in pancreatic cancer cell lines, based on previous reports where similar structures were employed in other tumor systems [29,30]. Intriguingly, after 48 h from the treatment, **C-1** and **C-2** were the most active in PANC-1, whereas **C-3** exerted a cytotoxic effect only in BXPC-3 cancer cells (Figure 2A,B). However, as illustrated in Figure S12, lower concentrations of trimers did not significantly affect cell viability.

Then, the different responses of pancreatic ductal adenocarcinoma cells to GEM were studied. First, as reported in previous studies [35], we observed that PANC-1 showed intrinsic chemoresistance to chemotherapy (Figure S13). To evaluate the potential effect as chemotherapy adjuvants of such derivates, the cell viability was studied when the trimers were combined with GEM. In the case of BXPC-3, the cell viability obtained in the presence of GEM did not change when it was combined with the trimers. Interestingly, in PANC-1, the combination of the trimers with GEM was able to overcome their intrinsic chemoresistance to this drug, especially when the chemotherapeutic was used at lower concentrations (0.5 and 4.5 µM) (Figure 2C and Figure S14). In particular, the combined treatment led to a synergistic enhancement of the cytotoxic effect, as shown by heat maps obtained with the Loewe Additivity model (Figure 2E) [31]. The synergistic effect is particularly relevant when the CPs were used at the concentration of 1000 µM and GEM at concentrations ranging from 0.5 to 20 µM. Then, to assess the specificity of these materials for cancer cells, similar experiments were also carried out in HaCaT cells, a non-cancerous cellular model. Interestingly, none of the three compounds exhibited toxicity in this cell line (Figure S15A) and did not enhance the cytotoxic activity of GEM (Figure S15B). These data suggest that these derivates are safe in non-tumor models and have therapeutic potential in the selective treatment of pancreatic cancer cells resistant to GEM.

### 3.3. The Combined Treatment Increased Apoptosis and Inhibited the mTOR Pathway in Pancreatic Cancer Cells

**Figure 3:**
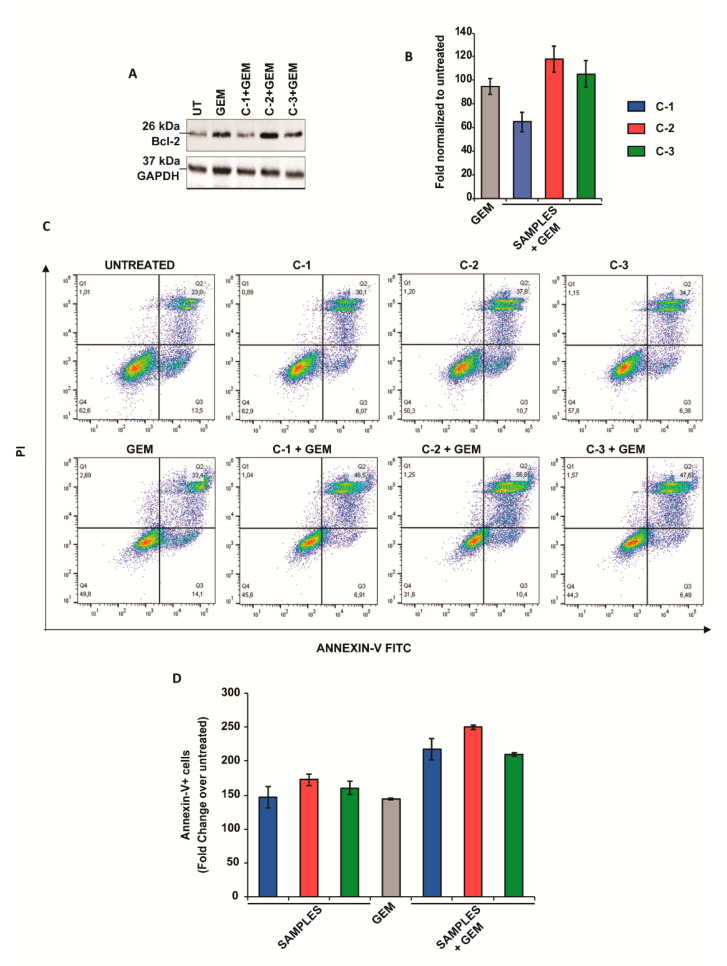
Apoptotic studies in pancreatic cancer cell lines. (**A**) Western blot analysis of Bcl-2 in PANC-1 cells after treatment with 1 mM **C-1, C-2, C-3**, and 4.5 μM GEM for 48 h. GAPDH protein level in the same extract was used as a control loading. (**B**) Densitometry of bands was performed using NIH Image J software and reported as fold change with respect to the untreated condition. The values reported are the mean of three independent experiments and were reported as mean ± SEM. (**C**) Cells were stained with Annexin V and propidium iodide (PI) and analyzed by flow cytometry. The percentage of cells in each group within the gated areas is indicated; the upper right panel represents cells undergoing late apoptosis, and the lower right panel represents cells undergoing early apoptosis. (**D**) Fold change in apoptosis. The values reported are the mean of two independent experiments and were reported as mean ±SEM.

To better understand the mechanism behind the antitumoral activity, some molecular markers related to apoptosis and cell growth in PANC-1 cancer cells were evaluated by Western blots (e.g., Bcl-2, p70S6K) and by Annexin-V assay. First, the effect of the trimers in Bcl-2 was assessed. This protein contributes to cancer formation and progression by promoting the survival of cancer cells and represents a canonical target for cancer therapy [36]. Interestingly, CPs did not change the levels of Bcl-2 when administered alone (Figure S16), however, a reduction of Bcl-2 was observed when PANC-1 cells were treated with **C-1** and GEM. However, no significant reductions in this protein were observed after the treatment with **C-2** or **C-3** in combination with GEM (Figure 3A,B).

Then, the cells were stained with fluorescein isothiocyanate (Annexin-V FITC) and propidium iodide (PI) and were analyzed by flow cytometry (Figure 3C,D). Interestingly, we observed an increase in Annexin-V fluorescence when the cells were treated with all three derivates and GEM, suggesting that the activation of apoptotic cell death may underlie the chemosensitivity observed.

Moreover, the activation of mTOR signaling after combined treatment was evaluated. It is worth mentioning that the inhibition in the mTOR signaling represents one of the mechanisms exploited by chemotherapy drugs to exert their antitumoral action [37]. Thus, when only GEM was administrated, chemoresistance in pancreatic cancer cells was induced. Remarkably, the administration of the therapeutic mixtures containing **C-1** or **C-2** or **C-3** and GEM, strongly abolished the chemoresistance mentioned before. Particularly, as discussed in below, they reduced the phosphorylation at Ser371 of p70S6 protein), indicating the downregulation of the mTOR pathway.

### 3.4. Reactive Oxygen Species (ROS) Generated by Coordination Complexes

**Figure 4:**
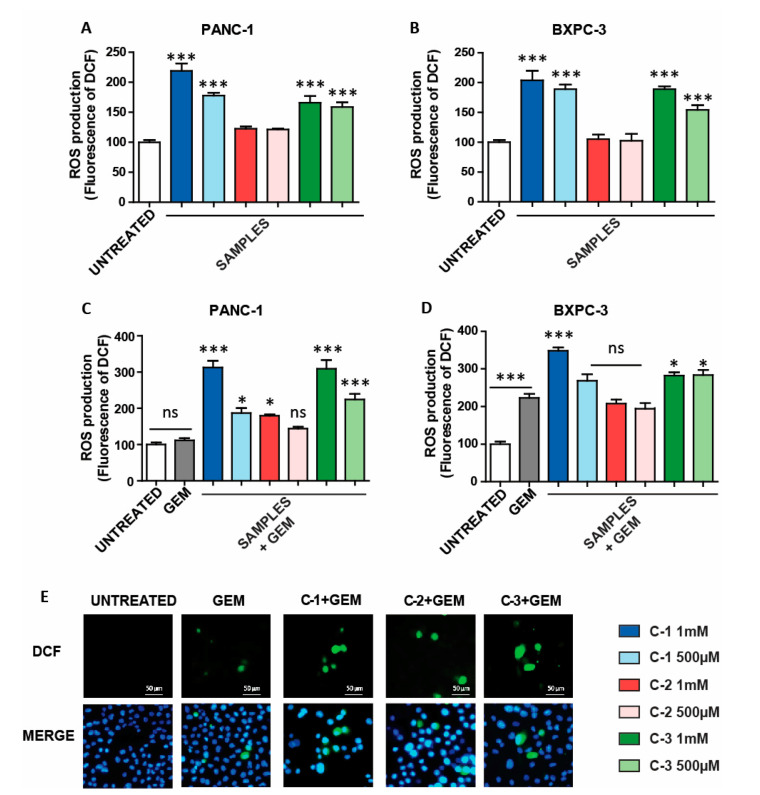
Reactive oxygen species (ROS) generated by coordination complexes. (**A**,**B**) ROS generation in pancreas cancer cell lines incubated with **C-1, C-2**, and **C-3** for 48 h. The DCF fluorescence (ROS production) of treated cells was normalized to untreated controls and reported as mean ±SEM. (**C**,**D**) ROS generation after combination therapy (incubation with GEM (4.5 µM) and **C-1, C-2**, and **C-3** for 48 h). The DCF fluorescence of treated cells was normalized to that of untreated controls and reported as mean ± SEM. Statistical analysis was performed using one-way ANOVA (each group vs. control). (*** *p* < 0.001, * *p* < 0.05). (**E**) Fluorescence images of PANC-1 cells untreated and treated with the combined therapy. DCF, corresponding to ROS production levels are shown in green, and the nuclei are labeled in blue by Hoechst staining.

Once the trimers were evaluated for their capabilities to synergistically increase the effect of GEM in PANC-1, the amount of ROS was evaluated [38], since they are usually generated in a variety of antitumoral systems. Thus, **C-1** to **C-3** were incubated with a standard ROS probe (DCFDA) at three different concentrations (1 µM, 100 nM, 1 nM) for 48 h. In this case, the fluorescent intensity increased in a concentration-dependent manner (Figure S17), highlighting the new trimers’ potential use as ROS generators. Interestingly, the ROS production by addition of Fe(OTs)_2_ was significantly lower compared to the results obtained with CPs at the concentration of 100 nM (Figure S18). Then, the new trimers’ activity was evaluated in two pancreatic cancer cell lines, PANC-1 and BXPC-3. Interestingly, **C-1** and **C-3** produced an enhancement on the ROS levels, whereas **C-2** did not provide any relevant effect compared to that of untreated samples (Figure 4A,B). This result suggests that **C-2** might be processed inside the cell differently, compared to **C-1** and **C-3**. Interestingly, the ROS production by addition of Fe(OTs)_2_ was significantly lower than in the case of **C-1** and **C-3** (Figure S19). These results suggest that iron-based CPs, such as **C-1** and **C-3**, can be employed to modify the oxidative status in pancreatic cancer cells, opening avenues to be exploited for therapeutic purposes.

Then, the activity of CPs was studied in combination with GEM, which is a standard agent used for the treatment of pancreatic cancer. It is important to point out that the cell lines (PANC-1 and BXPC-3) present different behavior against GEM. In particular, the presence of GEM induces a higher amount of ROS in BXPC-3 and reduces its viability more efficiently than in PANC-1 (Figure S20). In other words, as already mentioned above, PANC-1 presents chemoresistance to GEM, and for this reason, this cell line is often employed to assess novel therapeutics against pancreatic cancer. Thus, PANC-1 and BXPC-3 cancer cells were incubated with the three trimers and GEM for 48 h. In this case, a significant increase of ROS levels in both cell lines was observed when treated with **C-1** or **C-3** in the presence of GEM, compared to the ROS generated when treated only with GEM (Figure 4C,D). On the other hand, the combination **C-2** and GEM enhanced ROS only in PANC-1 cells. The ROS activity of the combined treatment was confirmed by fluorescent microscopy, where an increase in fluorescent signal was observed compared to that of the cells treated only with GEM (Figure 4E). Remarkably, when the iron salt and GEM were used, the ROS production was lower than in the cases where **C-1** and **C-3** were employed. This result highlights the advantage of the CPs evaluated herein compared to the iron salt Fe(OTs)_2_ (Figure S21). Then, similar experiments were also carried out in HaCaT cells. In this case, only **C-3** was able to increase ROS, and only weakly (Figure S22A). However, in the presence of GEM, a significant increase of ROS was observed in the case of **C-1** and **C-3**, as previously shown in pancreatic cancer cells (Figure S22B).

### 3.5. Combined Treatment Attenuates the mTOR Pathway in an ROS-Dependent Manner

**Figure 5:**
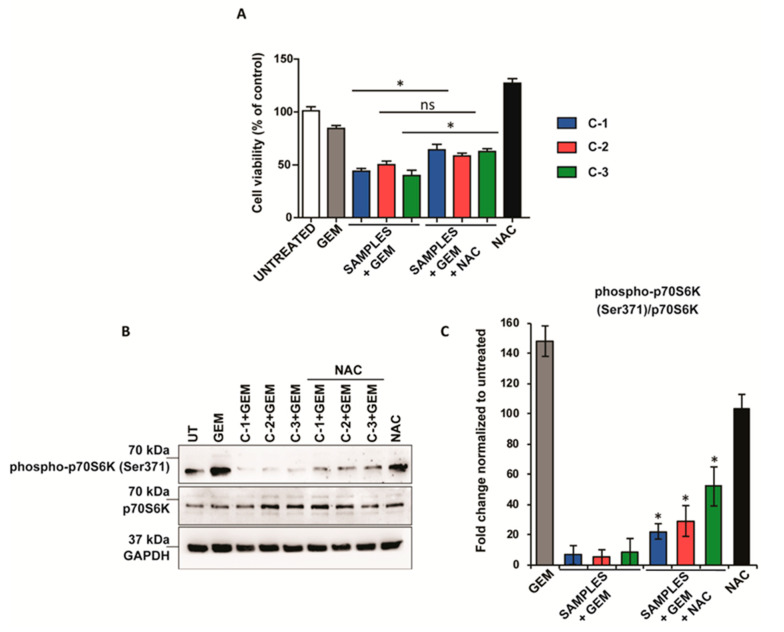
Combined treatment attenuates the mTOR pathway in an ROS-dependent manner. (**A**) PANC-1 cancer cell lines treated with 4.5 µM GEM, 500 µM NAC, and 1 mM **C-1** (blue), **C-2** (red), and **C-3** (green) for 48 h. Their viability was assessed with the alamarBlue test. The values of treated cells were normalized to that of untreated controls and reported as mean ± SEM. (**B**) Western blot analysis of phospho-p70SK6 and p70S6K of PANC-1 cells after treatment with 4.5 μM GEM, 500 μM NAC, and 1 mM of **C-1**, **C-2**, and **C-3** for 48 h. GAPDH protein level in the same extract was used as a control loading. (**C**) Densitometry of bands was performed using NIH Image J software and reported as fold change with respect to the untreated condition. The values reported are the mean of three experiments and were reported as mean ± SEM). Statistical analysis was performed using one-way ANOVA (C-1+GEM, C-2+GEM, C-3+GEM vs C-1+GEM+NAC, C-2+GEM+NAC, C-3+GEM+NAC)., * *p* < 0.05).

Finally, the effect of ROS generated by the therapeutic mixtures in the regulation of cell proliferation was assessed by the addition of the standard inhibitor of oxidative stress, *N*-acetyl cysteine (NAC), which does not affect cell viability (Figure S23). In this case, the effect on cell viability by the therapeutic mixtures was attenuated after NAC administration in PANC-1 cancer cells (Figure 5A). Intriguingly, when the mixture contained **C-2** and GEM, the removal of ROS had no significant effect on cell viability, suggesting that its synergistic effect was independent of oxidative stress. Accordingly, the increasing of ROS after the combined therapy was reduced after NAC treatment (Figure S24A). Furthermore, the phosphorylation at Ser371 of p70S6K, was also dramatically recovered by the addition of NAC (Figure 5B). These results highlight the role of ROS in the limitation of cancer cell growth mediated by the compounds described herein.

## 4. Discussion

In this work, we used a particularly interesting family of iron CPs, which is the chain-like polymeric Fe(II) system, with general formula {[Fe(Rtrz)3]X2}n, (Rtrz = substituted triazole; X = monovalent anion), which has been largely studied in a solid state. These Fe(II) systems present the physical phenomenon of spin-crossover, which is accompanied by a strong thermochromism and a magnetic transition between diamagnetic and paramagnetic behavior in the solid state, either as a powder or as nanoparticles. These CPs have not been studied very much in the aqueous solution, mainly due to their poor solubility. To overcome this limitation, we used in this report shorter polymeric chains (more precisely, trinuclear oligomers), achieving unprecedented water-soluble derivatives (**C-1**, **C-2**, **C-3**). Once these oligomers were synthesized and characterized by a multitude of techniques, their anticancer activities were studied for the first time. Remarkably, these derivatives were able to increase the activity of GEM in a pancreatic cancer cell line (PANC-1). Intriguingly, in BXPC-3, another pancreatic cancer cell line, the combined therapy did not enhance the chemotherapy effect, and the reduction observed was only due to GEM toxicity without any synergistic or additive effects. Furthermore, the specificity of our therapeutic systems was evaluated by assessing the activity of such derivates in HaCaT keratinocyte cells. In this case, none of the three compounds exhibited toxicity in this non-tumoral cell line and did not enhance the cytotoxic activity of GEM. These observations suggest that these derivates are safe in non-tumor models and have therapeutic potential in the selective treatment of pancreatic cancer cells resistant to GEM. The drastic difference between the pancreas cancer cell lines versus the non-tumoral model may be explained by, among other causes, acquired selective mutations, which lead to the regulation of different signaling pathways after GEM treatment. For example, chemoresistance of pancreatic cancer has been attributable to hyperactivation of MAPK [39], STAT-3, and NF-κB signaling pathways [40]. Moreover, the effects of GEM are mediated by transporters to cross extracellular or intracellular membranes, and oncogenic mutations occurring in solute carrier (SLC) and the ATP-binding cassette (ABC) superfamilies also have been linked to drug resistance in pancreatic cancer [41].

We investigated the molecular mechanisms underlying the activity of our CPs in pancreatic cancer cells. Interestingly, we observed that the CPs were able to attenuate chemoresistance in PANC-1 by inducing apoptosis and inhibiting mTOR signaling, which we detected through the phosphorylation of p70S6K, a classic downstream readout of this pathway. Curiously, although all three compounds were able to increase Annexin-v fluorescence when combined to GEM, the reduction of Bcl-2 protein, a classical inhibitor of apoptosis, was observed only when the therapeutic mixture contained C-1. This partial discrepancy could be explained in part because Bcl-2′s role in apoptosis is ambiguous. Indeed, Bcl-2 can also act as an apoptotic inducer blocking Bax/Bak oligomerization [42] and plays a role in other non-canonical functions including mitochondrial metabolism and biogenesis and autophagy regulation [43]. Another explanation could be that even if the translocation of phosphatidylserine is produced after the treatment, a process that is usually associated with the early stage of apoptosis, this does not result in the complete activation of the intrinsic apoptosis pathway and the formation of apoptosomes, which precedes caspase’s activation [44].

ROS play a key role in various cellular processes, including proliferation, growth, apoptosis, and migration [45]. Importantly, ROS levels are frequently increased in cancer cells because of their high metabolic activity, mitochondrial dysfunction, and hyper-activation of oncogenes [46]. However, while ROS facilitate carcinogenesis and cancer progression with mild-to-moderate elevated levels, their excessive production may damage cancer cells dramatically through the modification of lipids, proteins, or DNA and lead to cell death [28]. Hence, ROS signaling and the antioxidant program play key roles in the response to chemotherapy and represent important targets to overcome drug-resistance [47].

In this study, the effect of our derivates on ROS metabolism in pancreatic cancer cells was investigated. Once we established that such structures were able to increase ROS production, both alone and in combination with GEM, we studied whether the cytotoxic effect of the combined therapy was due, at least in part, to the CPs’ capacity to enhance ROS levels. To unveil this, experiments using the ROS scavenger NAC were performed, which showed that NAC could recover cell viability and the mTOR pathway affected by trimers and GEM.

Overall, these data allow us to speculate that both apoptosis induction and mTOR inhibition, resulting after combined therapy, might contribute to the synergistic reduction of cell viability observed in pancreatic cancer cells, and also that the cytotoxic effect is due to the increase in oxidative stress.

## 5. Conclusions

In summary, we have demonstrated that CPs can be used as adjuvants to enhance synergistically the activity of GEM in pancreatic cancer cell lines, which present intrinsic resistance to this chemotherapeutic through modulation of ROS levels. Interestingly, in contrast to that observed in PANC-1, the combined therapy did not enhance the chemotherapy effect either in BXPC-3, a pancreatic cancer cell line not resistant to GEM, nor in HaCaT keratinocyte cells, and the reduction of cell viability was only due to GEM toxicity without any synergistic or additive effect.

Furthermore, we studied the signaling pathways and molecular mechanisms underlying the chemosensitivity to GEM. We observed that the combined treatment increased apoptosis and reduced the phosphorylation of p70S6k, a well-established functional readout of the mTOR pathway involved in cell growth.

Finally, to unveil whether the increased ROS detected after combined therapy could have a role in the CPs’ therapeutic effect, we performed functional studies using NAC, a standard inhibitor of oxidative stress. Notably, the addition of NAC significantly reverted the reduction of cell viability and the mTOR pathway observed after combined therapy, suggesting the CPs’ effect was, at least in part, due to exacerbated ROS production in PANC-1 GEM-resistant cell lines.

## Data Availability

Data are contained within the article. The raw data of blots are available from the corresponding author upon reasonable request.

## Supplementary Materials

The following are available online at https://www.mdpi.com/2076-3921/10/1/66/s1, Figure S1: Scheme of the reaction of the ligands TE-Tria and PE-Tria, Figure S2: ^1^H NMR (400 MHz, DMSO-d6, 298 K) of TE-Tria, Figure S3: Infrared spectrum of TE-Tria, Figure S4: ^1^H NMR (400 MHz, DMSO-d6, 298 K) of the PE-Tria, Figure S5: Infrared spectrum of PE-Tria, Figure S6: UV-vis spectra of (a) **C-1** in water, (b) **C-1** in PBS, (c) **C-2** in water, (d) **C-2** in PBS, (e) **C-3** in water, and (f) **C-3** in PBS, Figure S7: Solution of **C-1, C-2**, and **C-3** (10^−2^ M) in water initially and aged for 24 and 48 h (a) with ascorbic acid and (b) without ascorbic acid, Figure S8: Infrared spectrum of **C-1**, Figure S9: Infrared spectrum of **C-2**, Figure S10: Infrared spectrum of **C-3**, Figure S11: Powder X-ray Diffraction of **C-1**, **C-2**, and **C-3**, Figure S12: Cell viability studies in pancreas cells, Figure S13: Dose response curve of GEM, Figure S14: Dose response studies in pancreas cancer cells, Figure S15: Cell viability studies in human keratinocytes, Figure S16 CP administered alone does not affect Bcl-2 protein level, Figure S17: DCF fluorescence generated by coordination complexes, Figure S18: DCF fluorescence generated by salt and hydrogen peroxide, Figure S19: ROS generation in pancreas cancer cell lines, Figure S20: Different responses to gemcitabine in pancreatic cancer cells, Figure S21: ROS generation in pancreatic cancer cell lines after combined treatment, Figure S22: ROS generation in human keratinocytes, Figure S23: Cell viability studies in pancreas cancer cells, Figure S24: Combined treatment attenuates the mTOR pathway in an ROS-dependent manner.
