# Supplementary material for: Water Soluble Iron-Based Coordination Trimers as Synergistic Adjuvants for Pancreatic Cancer
Source: Antioxidants (Basel). 2021 Jan 7;10(1):66. doi: 10.3390/antiox10010066 (PMC7825762; doi:10.3390/antiox10010066)
Supplement: Supplementary file 1 [file antioxidants-10-00066-s001.pdf]

Supplementary Material for

Water soluble Iron-Based Coordination Trimers as Synergistic Adjuvants for Pancreatic Cancer

Marco Cordani [1], Esther Resines-Urien [1], Arturo Gamonal [1], Paula Milán-Rois [1], Lionel Salmon [2], Azzedine Bousseksou [2], Jose Sanchez Costa [1,*] and Álvaro Somoza [1,*]

[1] IMDEA Nanociencia, Faraday 9, Ciudad Universitaria de Cantoblanco, 28049 Madrid, Spain; marco.cordani@imdea.org (M.C.); esther.resines@imdea.org (E.R.-U.); arturo.gamonal@imdea.org (A.G.); paula.milan@imdea.org (P.M.-R.)
[2] Laboratoire de Chimie de Coordination, UPR8241, 205 Route de Narbonne, CEDEX 4, 31077 Toulouse, France; lionel.salmon@lcc-toulouse.fr (L.S.); azzedine.bousseksou@lcc-toulouse.fr (A.B.)
* Correspondence: jose.sanchezcosta@imdea.org (J.S.C.); alvaro.somoza@imdea.org (Á.S.); Tel.: +34-912-998-848 (J.S.C.); +34-912-998-856 (Á.S.)

Contents

I. Synthesis and characterization of the ligands (TE-Tria and PE-Tria) and coordination polymers (C-1, C-2 and C-3).
II. Supporting figures

I. Synthesis and characterization of the ligands (TE-Tria and PE-Tria) and coordination polymers (C-1, C-2 and C-3).

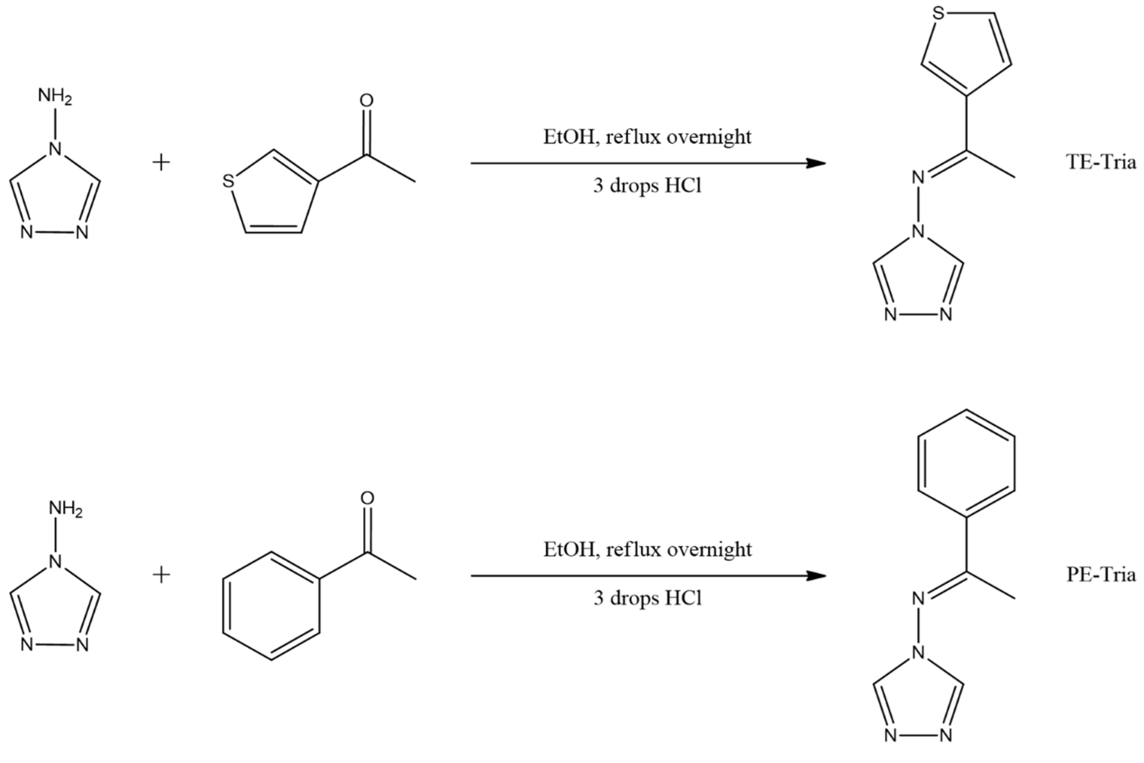

Figure S1. Scheme of the reaction of the ligands TE-Tria and PE-Tria.

Both ligands [(E)-N-(1-(thiophen-2-yl)ethylidene)-4H-1,2,4-triazol-4-amine (TE-Tria) and (E)-N-(1-phenylethylidene)- 4H-1,2,4-Triazol-4-amine] (PE-Tria) were carried out using the following an standard procedure for condensation. A mixture of 4-amino-1,2,4- triazole (1.968 g, 24 mmol) and the corresponding ketone (TE-Tria: 3-acetylthiophene (3.000 g, 24 mmol); PE-Tria: acetophenone (2.8 mL, 24 mmol)) in ethanol (50 mL) with 3 drops of HCl (acting as a catalyst of the reaction) was refluxed overnight. The solvent was eliminated and the resultant white solid (TE-Tria: 4.109 g, 92%; PE-Tria: 3.975 g, 89%) was thoroughly washed with ethanol.

TE-Tria

^{1}H NMR (400 MHz, DMSO-d$_6$, δ): 8.76 (s, 2H), 8.43 (d, *J*=2.8 Hz, 1H), 7.71 (dd, J_1=5.1 Hz; J_2=2.8 Hz, 1H), 7.66 (d, *J*=5.1 Hz, 1H), 2.35 (s, 3H) ppm. FTIR (cm^{-1}): 3299 (b), 3137 (m), 3088 (s), 2938 (w), 1773 (w), 1667 (w), 1597 (m), 1520 (m), 1499 (s), 1449 (m), 1419 (w), 1390 (w), 1366 (m), 1323 (w), 1312 (w), 1294 (w), 1273 (s), 1209 (m), 1169 (s), 1069 (w), 1077 (w), 1060 (s), 966 (m), 940 (m), 891 (m), 872 (m), 836 (m), 813 (s), 773 (m), 713 (m), 665 (m), 641(s), 621 (s), 598 (w), 526 (w), 463 (m), 446 (m), 416 (s) (See Figure S2 and S3)

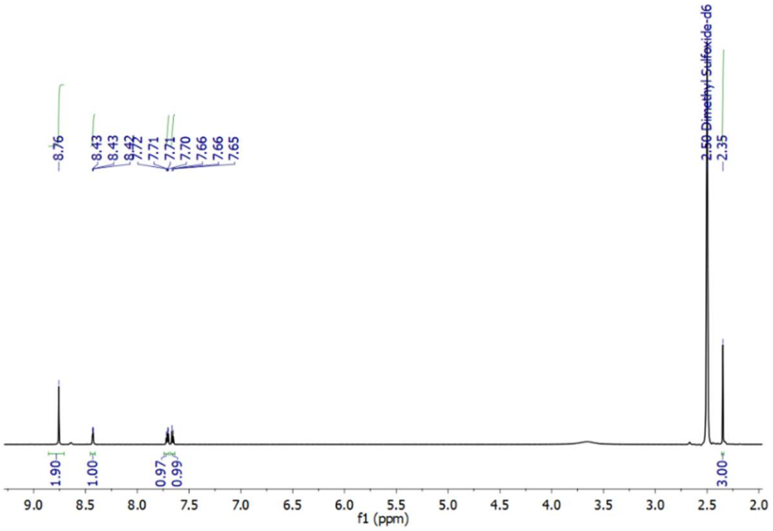

Figure S2. ^{1}H NMR (400 MHz, DMSO-d$_6$, 298 K) of TE-Tria.

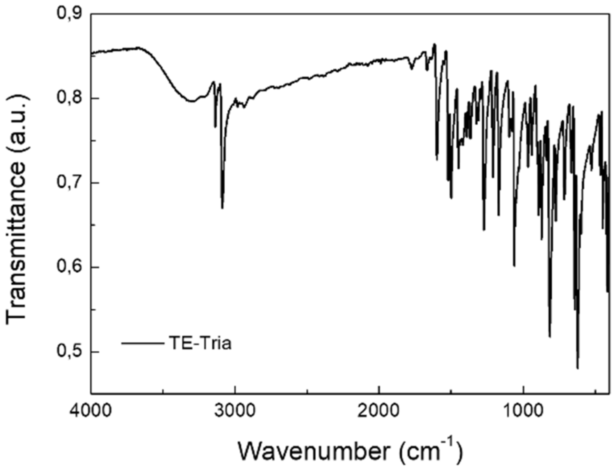

Figure S3. Infrared spectrum of TE-Tria.

PE-Tria

^{1}H NMR (400 MHz, DMSO-d$_6$, δ): 8.77 (s, 2H), 7.99 (d, *J*=7.1 Hz, 2H), 7.61 (d, *J*=7.3 Hz, 1H), 7.54 (t, *J*=7.4 Hz, 2H), 2.38 (s, 3H) ppm. FTIR (cm^{-1}): 3295 (b), 3124 (m), 2923 (w), 1682 (w), 1611(m), 1591 (w), 1572 (w), 1518 (w), 1489 (m), 1442 (m), 1369 (m), 1335 (m), 1306 (w), 1282 (s), 1076 (m), 1057 (s), 1026 (w), 978 (w), 961 (w), 937 (w), 854 (m), 766 (s), 689 (s), 622 (s), 595 (m), 597 (m), 470 (w), 448 (m).
(See Figure S4 and S5)

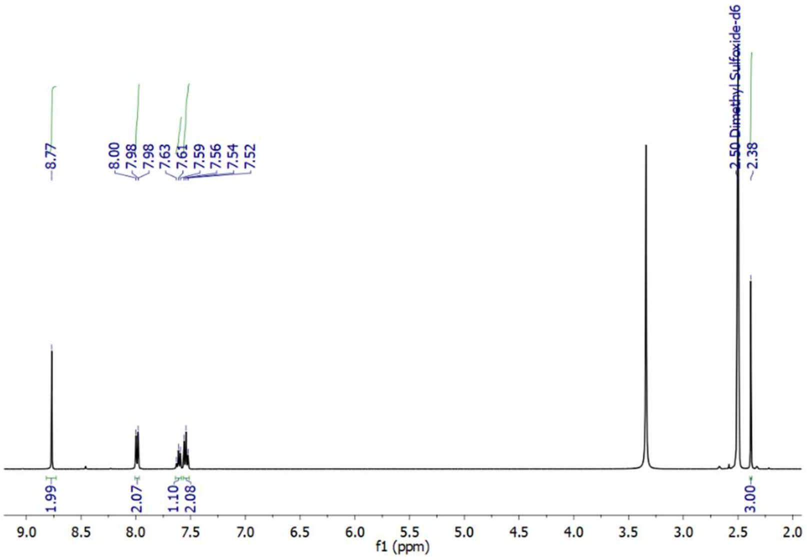

Figure S4. ^{1}H NMR (400 MHz, DMSO-d$_6$, 298 K) of the PE-Tria.

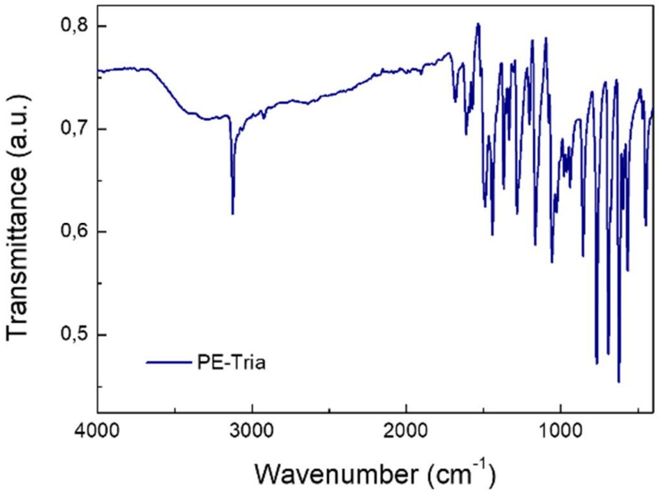

Figure S5. Infrared spectrum of PE-Tria.

Synthesis of the coordination complexes (**C-1**), (**C-2**) and (**C-3**) was carried out mixing Fe(II) tosylate (0.03 mmol, 16.9 mg) with the corresponding triazole (TE-Tria: 0.1 mmol; 19.2 mg;

NH_2-trz: 0.1 mmol, 8.2 mg; PE-Tria: 0.1 mmol, 18.6 mg) and ascorbic acid which prevents oxidation of the iron (II) cation (approx. 3 mg) in 10 mL of water. The reaction was stirred for 10 minutes until complete dissolution and filtered. The obtained trinuclear triazole-based CP with general structure $[Fe_3(RN\text{-}trz)_6(H_2O)_6]$ have been confirmed by ESI-MS spectroscopy. ESI-MS for $[Fe_3(NH_2\text{-}trz)_6(OTs)_4]^{2+}$ (C-2) calcd m/z: 678.0560; found m/z: 678.0578.

Both C-1 and C-3 present this same peak at 678.0575 and 678.0572, respectively. This result correspond to the hydrolyzation of the CPs to the primary amine coordination polymer (C-2) [1]by ESI-MS. In both cases, the product of decomposition of the ligand can also be observed:

ESI-MS for $[Acetylthiophene+H]^+$ ($C_6H_6OS+H^+$) calcd m/z:127.0212; found m/z: 127.0207.

ESI-MS for $[Acetophenone+H]^+$ ($C_8H_8O+H^+$) calcd m/z: 121.0648; found m/z: 121.0639

UV-Vis study of complexes C-1, C-2 and C-3

The UV-Vis study was carried out in water and PBS for the three different CP in concentrations 10^{-5} M. For this purpose, the CPs solutions (10^{-3}M) were prepared and kept at 37°C, replicating the incubation conditions. Then, to not saturate the UV spectra, the solutions were diluted to 10^{-5}M and were measured at three different times: t0, 24 and 48 hours. In all cases, the spectra show the same pattern initially and after 24-48 hours. As can be observed in Figure S6 the spectra show the characteristic bands assigned to the ligand. It is noteworthy the enhancement of the maxima intensity without any shifts of the band, and also the absence of new bands. This shows the stability of the complexes in these conditions. This data is in agreement with Figure S7, which shows the solution of the complexes at concentrations 10^{-2} M in water with and without ascorbic acid (the presence of this acid prevents the oxidation of the iron (II) center). The absence of color in the solutions with ascorbic acid is a proof of their stability after 48 hours. As a control, a solution without ascorbic acid was made and after only 24 hours, a yellow color has appeared, indicating the oxidation of iron (II) to iron (III).

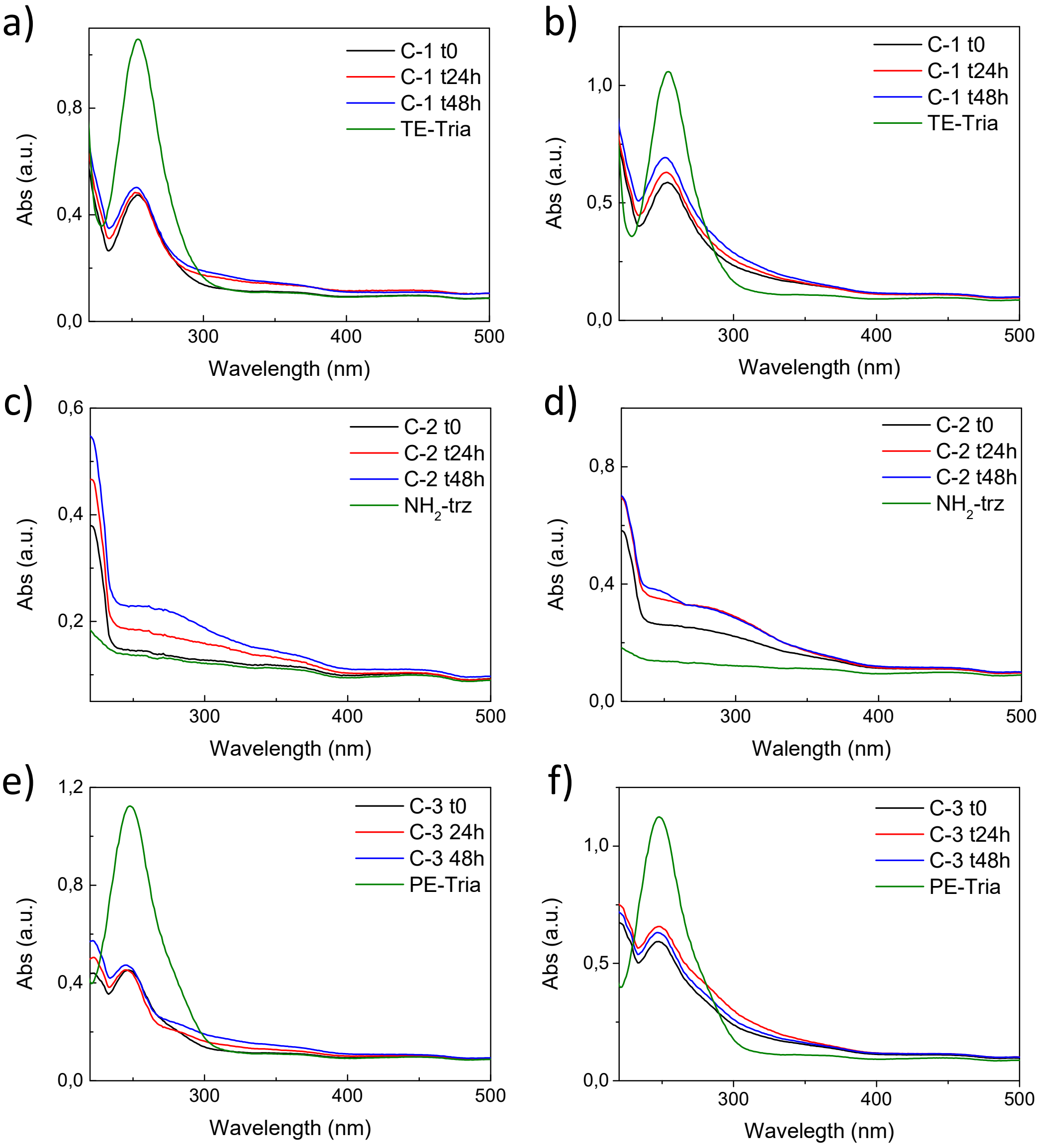

Figure S6. UV-vis spectra of a) **C-1** in water, b) **C-1** in PBS, c) **C-2** in water, d) **C-2** in PBS, e) **C-3** in water and f) **C-3** in PBS.

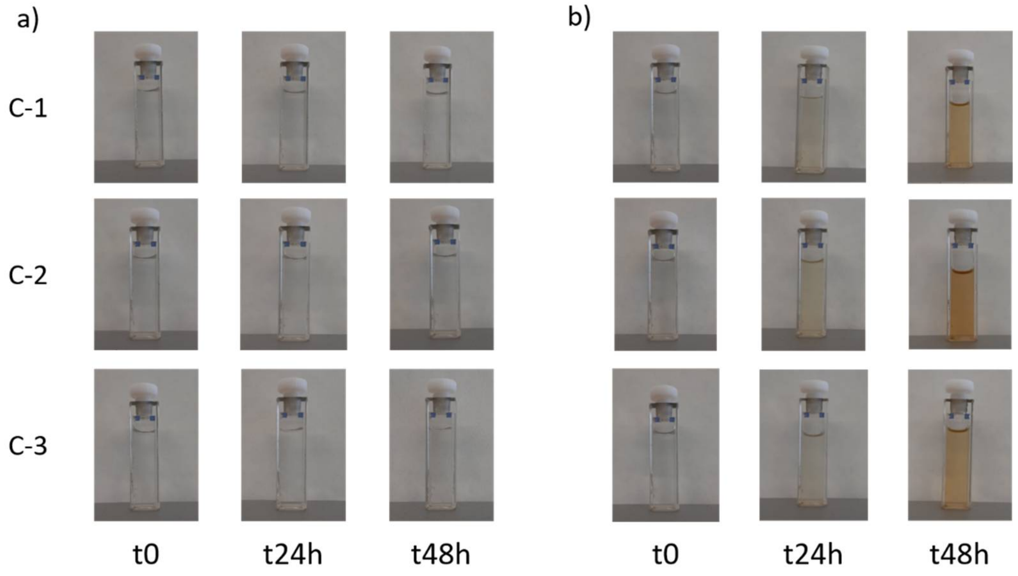

Figure S7. Solution of **C-1**, **C-2** and **C-3** (10^{-2} M) in water initially and aged for 24 and 48 hours a) with ascorbic acid and b) without ascorbic acid.

The solution was evaporated until the coordination polymers precipitate and those were characterized by FTIR. The bands observed from the ligands IR spectra are maintained after coordination, although slightly shifted. The C=N stretching mode from the imine group can be seen at 1530 and 1529 cm^{-1} for **C-1** and **C-3**, respectively. A sharp band, corresponding to the NH stretching mode is observed at 3293 cm^{-1} for **C-2**. The presence of the *p*-toluenesulfonate counterion is evidenced by singlets at 1170 cm^{-1}, 680 cm^{-1} and 563 cm^{-1} and a doublet (centered around 1032 and 1008 cm^{-1}), which are linked to the C-C bending and stretching modes in the phenyl group and the SO_3 group symmetric stretching.

C-1: FTIR (cm^{-1}): 3463 (w), 3290 (w), 3059 (w), 1646 (w), 1594 (w), 1530 (m), 1495 (w), 1413 (w), 1395 (w), 1368 (w), 1279 (w), 1172 (s), 1121 (s), 1090 (m), 1032 (s), 1008 (s), 905 (w), 876 (w), 812 (m), 792 (m), 681 (s), 625 (m), 563 (s), 467 (w).

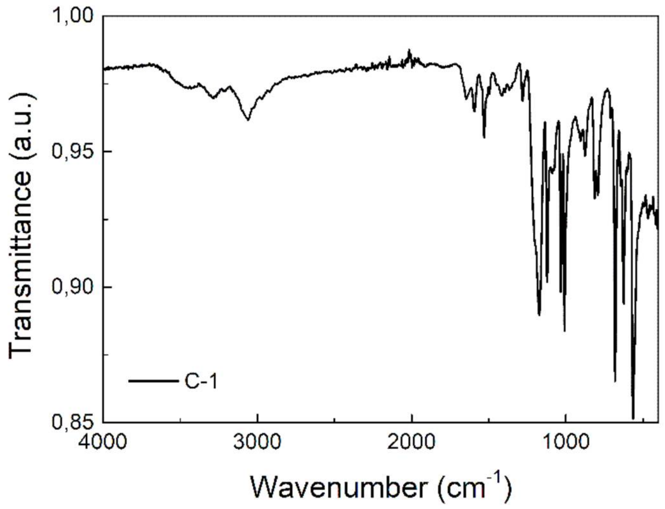

Figure S8. Infrared spectrum of **C-1**.

C-2: FTIR (cm^{-1}): 3441 (w), 3293 (m), 3210 (w), 3210 (m), 3062 (m), 1631 (m), 1546 (w), 1496

(w), 1449 (w), 1396 (w), 1170 (s), 1122 (s), 1098 (m), 1033 (s), 1008 (s), 881 (w), 813 (m), 681 (s), 623 (s), 563 (s).

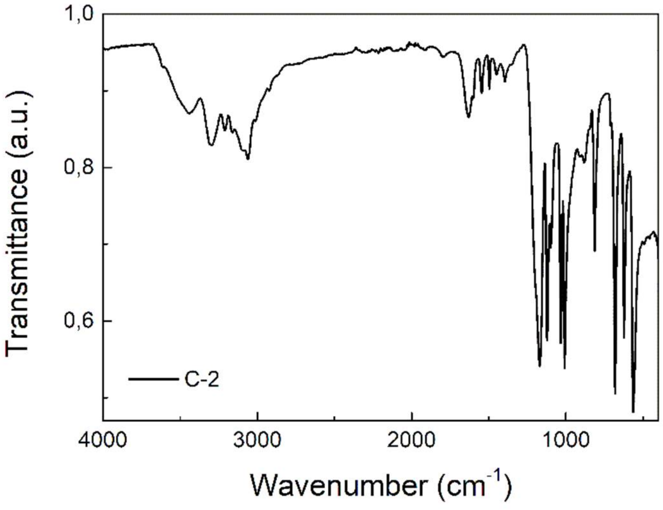

Figure S9. Infrared spectrum of **C-2**.

C-3: FTIR (cm^{-1}): 3273 (w), 3062 (m), 2965 (w), 2922 (w), 1796 (w), 1765 (w), 1681 (m), 1596 (m), 1570 (w), 1529 (m), 1496 (w), 1446 (m), 1362 (w), 1306 (w), 1294 (w), 1265 (w), 1175 (s), 1119 (s), 1072 (m), 1030 (s), 1008 (s), 919 (w), 814 (m), 763 (m), 679 (s), 625 (s), 590 (w), 562 (s), 492 (w).

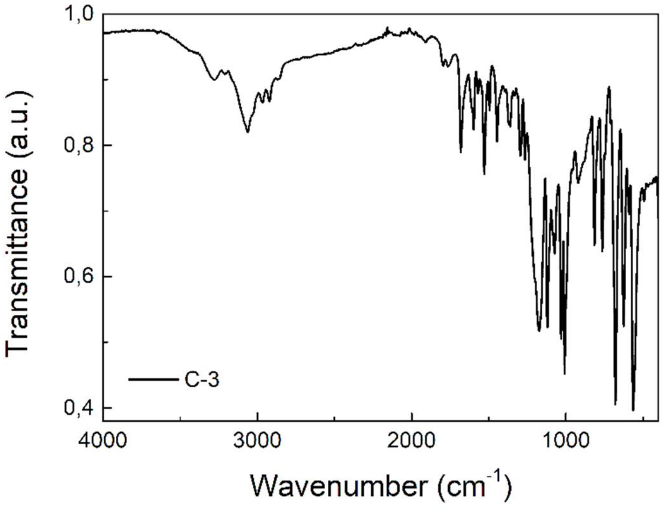

Figure S10. Infrared spectrum of **C-3**.

While every attempt to obtain single crystals of **C-1, C-2** and **C-3** has been unsuccessful, Powder X-ray Diffraction (PXRD) measurements were carried out to understand further the structure of the three coordination polymers (See Figure S11). The principal conclusion is that the three PXRD patterns are different, which confirms the structural diversity that can be associated with the different species.

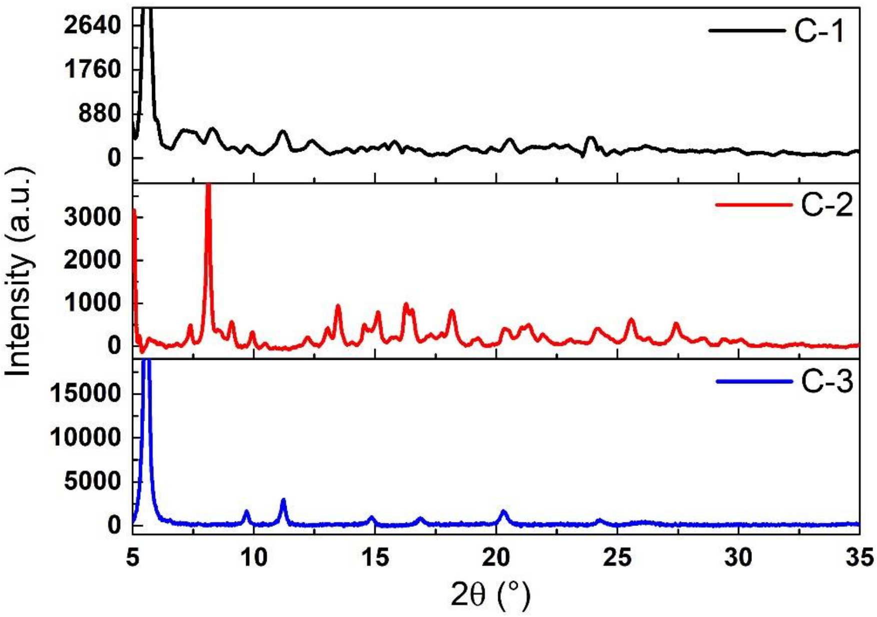

Figure S11. Powder X-ray Diffraction of **C-1**, **C-2** and **C-3.**

II. Supplementary figures

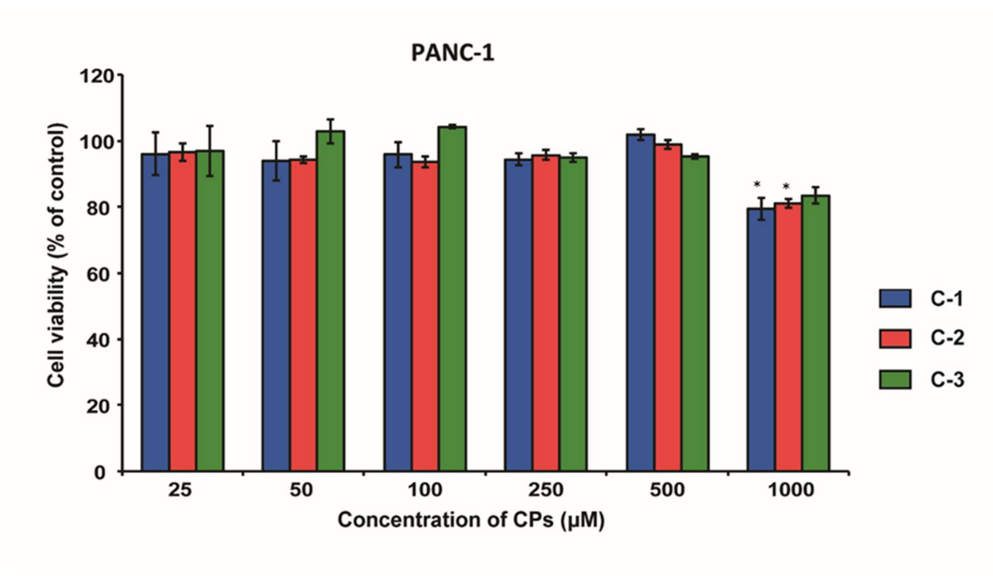

Figure S12. Cell viability studies in pancreas cells. PANC-1 pancreatic ductal adenocarcinoma cells were incubated with the indicated concentration of **C-1**, **C-2** and **C-3** for 72 hours and their viability assessed with the alamarBlue test. The values of treated cells were normalized to that of untreated controls and reported as mean ± SD. Statistical analysis was performed using one-way ANOVA (each group vs. untreated control). (* $p < 0.05$).

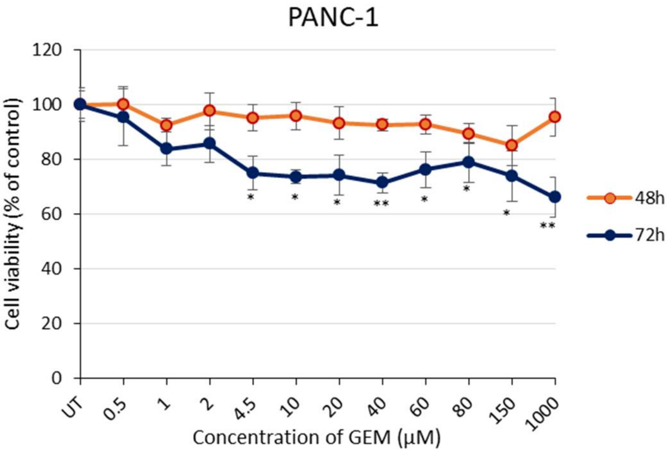

Figure S13. Dose Response Curve of GEM. PANC-1 pancreatic ductal adenocarcinoma cells were seeded in 96-well plates, incubated overnight, and treated with GEM at the indicated concentrations for 48 and 72 hours. Their viability assessed with the alamarBlue test. The values of treated cells were normalized to that of untreated controls and reported as mean ± SD. Statistical analysis was performed using one-way ANOVA (each group vs. untreated control). (** $p < 0.01$, * $p < 0.05$).

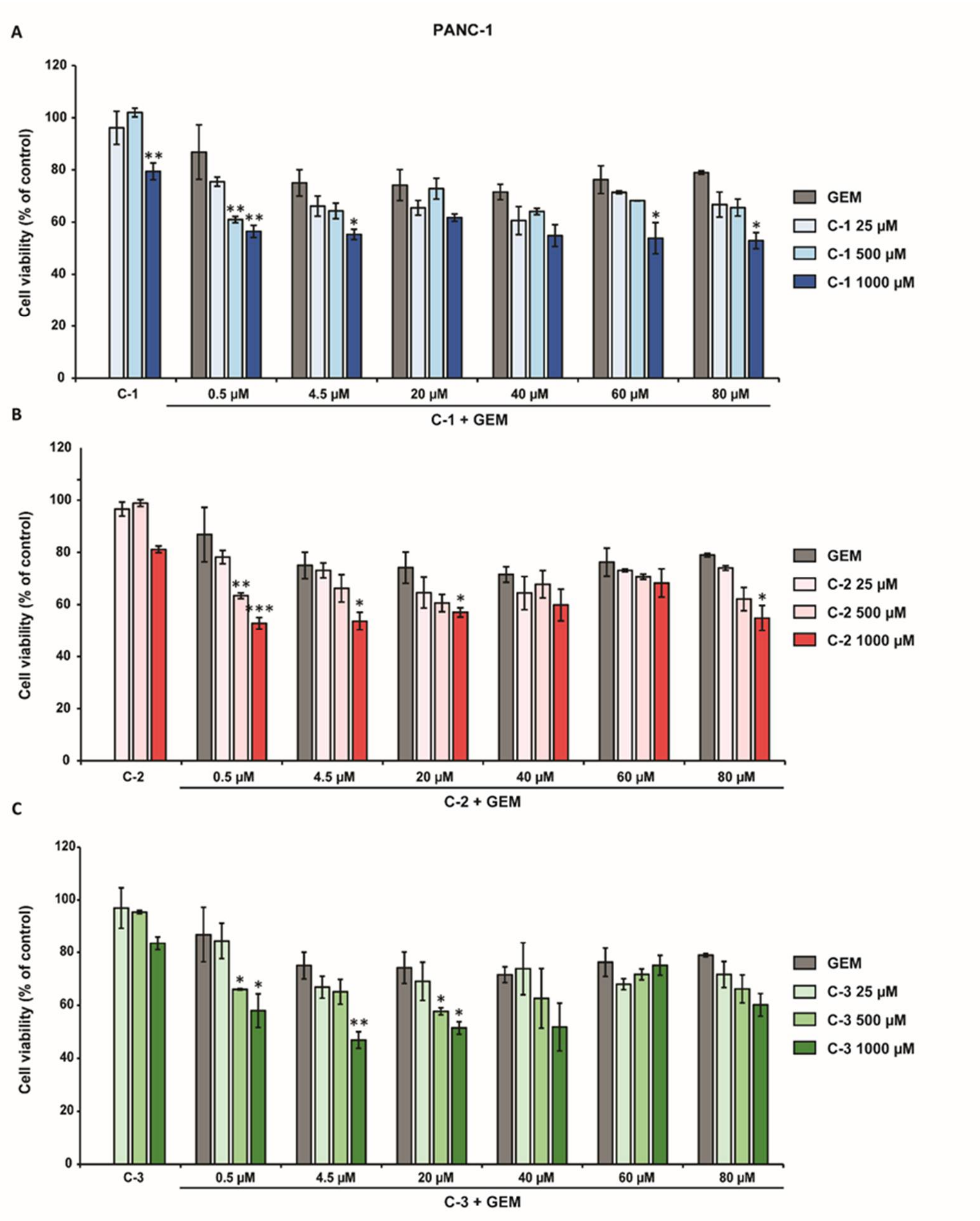

Figure S14. Dose response studies in pancreas cancer cells. Cell viability studies in PANC-1 pancreatic ductal adenocarcinoma cells (A-C) treated with **C-1**, **C-2** and **C-3** at the indicated concentrations and in combination with 0.5, 4.5, 20, 60 or 80 μM GEM for 72 hours. The values of treated cells were normalized to the untreated controls and reported as mean ± SEM. Statistical analysis was performed using one-way ANOVA (**C-1**+GEM, **C-2**+GEM, **C-3**+GEM vs GEM). (*** $p < 0.001$, ** $p < 0.01$, * $p < 0.05$).

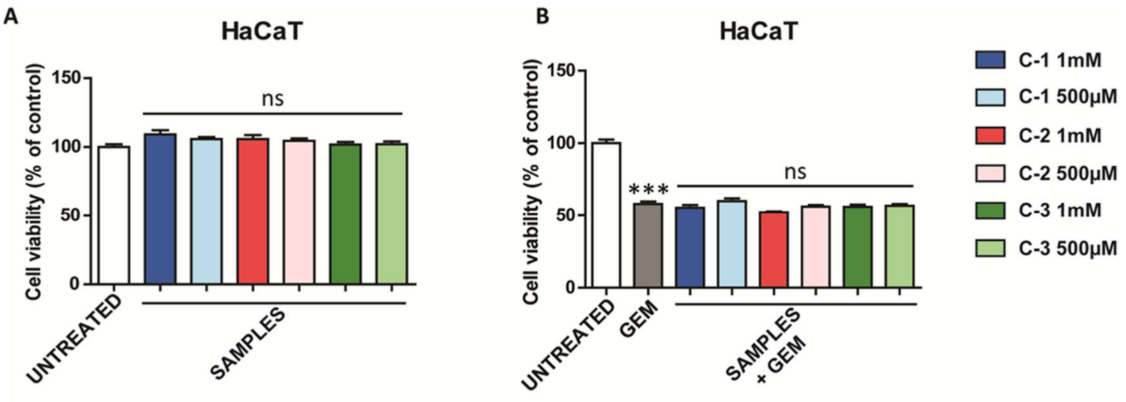

Figure S15. Cell viability studies in human keratinocytes. HaCaT cells were incubated with **C-1**, **C-2** and **C-3** alone (A) and in combination with 4.5 µM GEM (B) for 48 hours and their viability assessed with the alamarBlue test. The values of treated cells were normalized to that of untreated controls and reported as mean ± SD. Statistical analysis was performed using one-way ANOVA (each group vs. control). (*** $p < 0.001$).

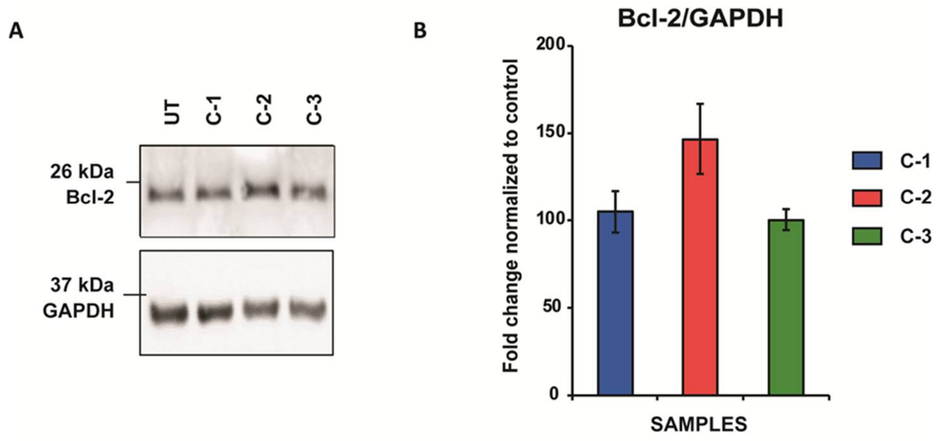

Figure S16. CP administered alone do not affect Bcl-2 protein level. A) Western blot analysis of Bcl-2 in PANC-1 cells after treatment with **C-1**, **C-2**, **C-3** for 48 hours. GAPDH protein level in the same extract was used as a control loading. B) Densitometry of bands was performed using NIH Image J software and reported as fold change respect to the untreated condition. The values reported are the mean of three experiments and were reported as mean ± SEM.

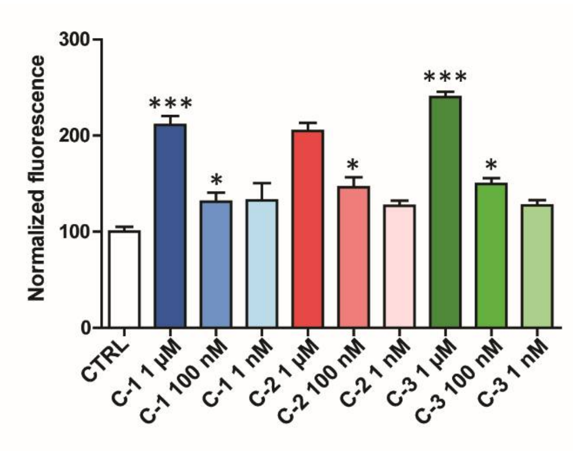

Figure S17. DCF fluorescence generated by coordination complexes. The complexes **C-1**, **C-2** and **C-3** were mixed with 1 µM DCF in water for 48 hours. The fluorescence was normalized to that of untreated controls and reported as mean ± SD. Statistical analysis was performed using one-way ANOVA (each group vs. control). (*** $p < 0.001$, * $p < 0.05$).

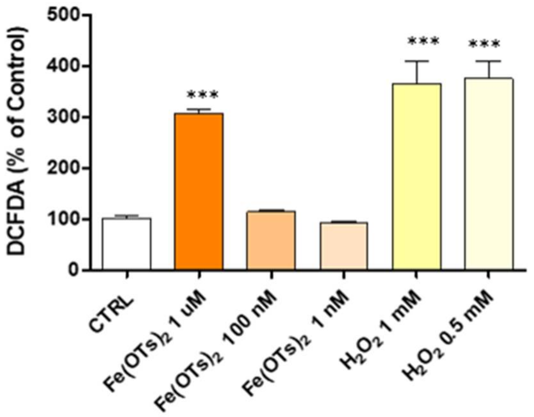

Figure S18. DCF fluorescence generated by salt and hydrogen peroxide. Salt and hydrogen peroxide were mixed to 1 µM DCF in water for 48 hours. DCF fluorescence intensity was measured by a multimode plate reader. The DCF fluorescence resulting was normalized to that of untreated controls and reported as mean ± SD. Statistical analysis was performed using one-way ANOVA (each group vs. control). (*** $p <$ 0.001).

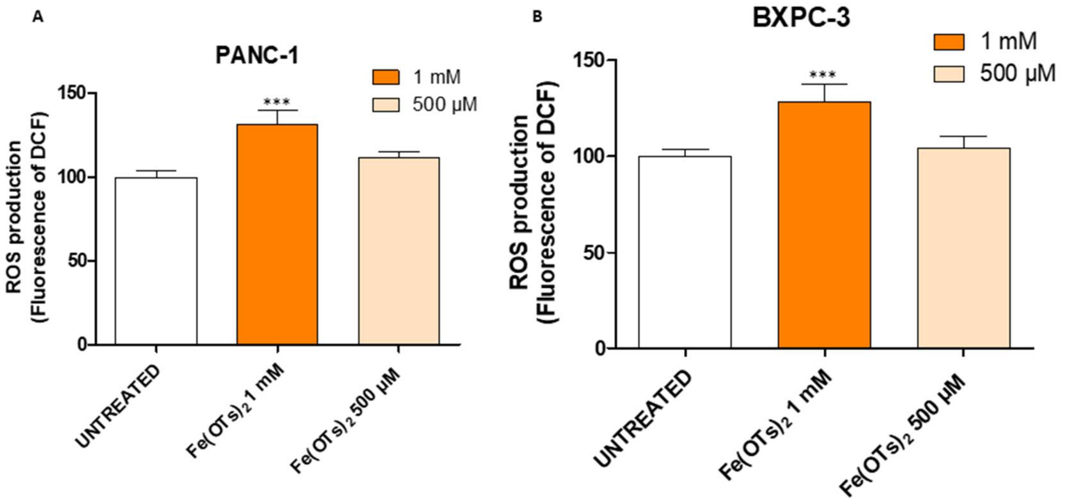

Figure S19. ROS generation in pancreas cancer cell lines. A-B) The indicated pancreas cancer cells were seeded in 96-well plates, incubated with $Fe(OTs)_2$ (Iron salt) for 48 hours at the concentrations indicated. DCF fluorescence intensity, corresponding to ROS production level, measured by a multimode plate reader. The DCF fluorescence of treated cells was normalized to that of untreated controls and reported as mean ± SD. Statistical analysis was performed using one-way ANOVA (each group vs. control). (*** $p < 0.001$).

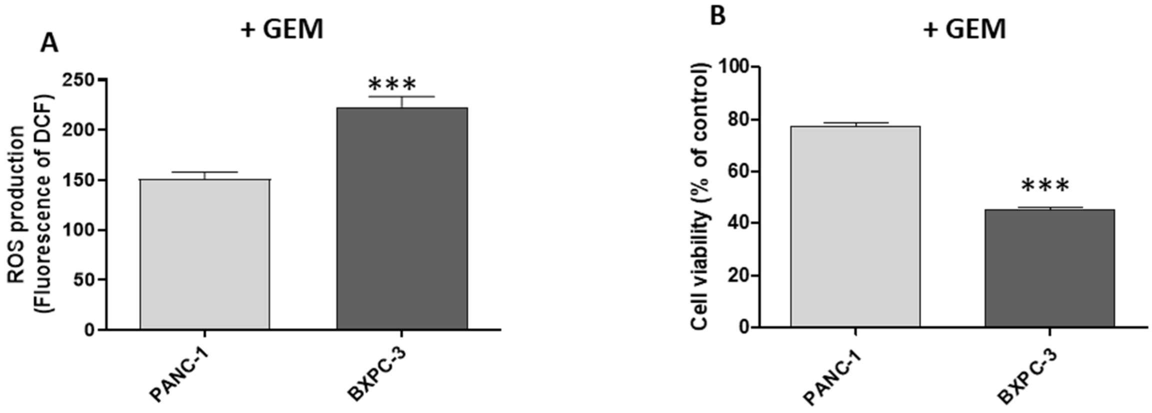

Figure S20. Different responses to Gemcitabine in pancreatic cancer cells. A) Pancreatic cancer cell lines were seeded in 96-well plates and incubated with GEM (4.5 μM) for 48 hours. The DCF fluorescence of treated cells was normalized to that of untreated controls and reported as mean ± SD. Statistical analysis was performed using one-way ANOVA (each group vs. control). (*** $p < 0.001$). B) Pancreatic cancer cell lines were seeded in 96-well plates and incubated with GEM (4.5 μM) for 48 hours. Statistical analysis was performed using one-way ANOVA (each group vs. control). (*** $p < 0.001$).

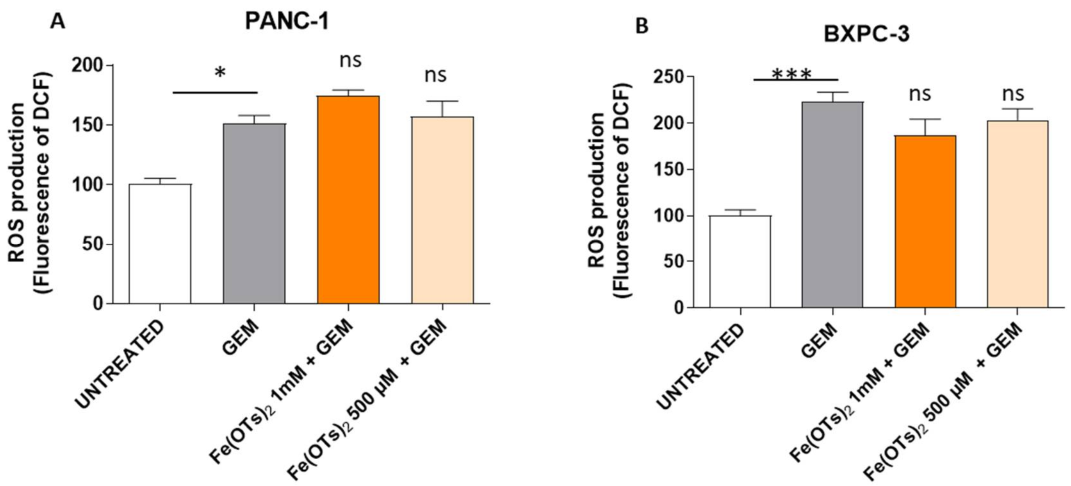

Figure S21. ROS generation in pancreatic cancer cell lines after combined treatment. Pancreatic cancer cells (A: Panc-1, B: BxPC-3) were incubated with 4.5 µM GEM and $Fe(OTs)_2$ for 48 hours. The DCF fluorescence of treated cells was normalized to that of untreated controls and reported as mean ± SD. Statistical analysis was performed using one-way ANOVA (each group vs. control). (*** $p < 0.001$, * $p < 0.01$).

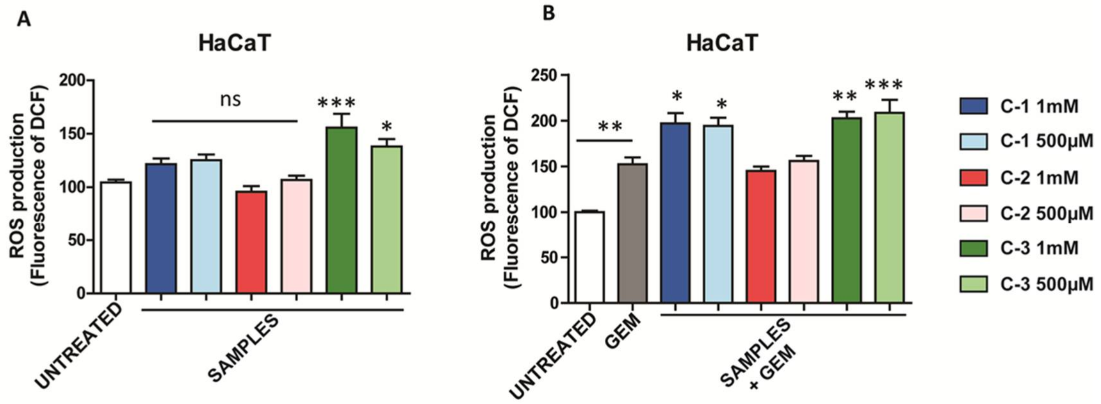

Figure S22. ROS generation in human keratinocytes. HaCaT cells were incubated with **C-1**, **C-2** and **C-3** alone (A) and in combination with 4.5 µM GEM (B) for 48 hours. ROS generation was measured as DCF fluorescence as reported in the methods. The values of treated cells were normalized to that of untreated controls and reported as mean ± SD. Statistical analysis was performed using one-way ANOVA (each group vs. control). (*** $p < 0.001$, ** $p < 0.01$, * $p < 0.05$).

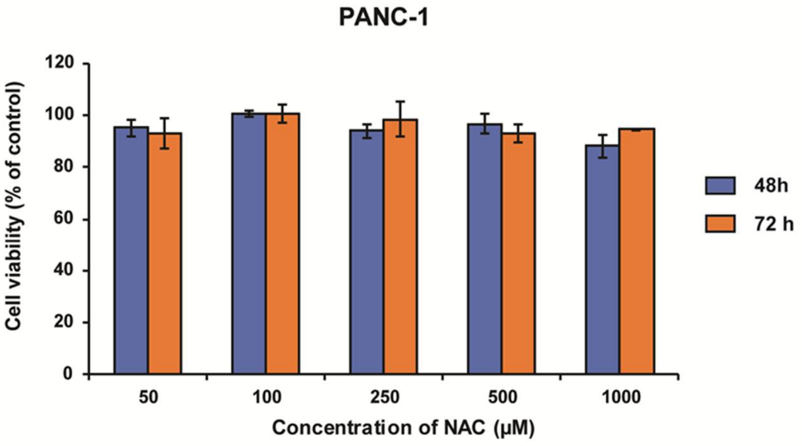

Figure S23. Cell viability studies in pancreas cancer cells. PANC-1 cancer cells were incubated with NAC at the indicated concentrations for 48 and 72 hours and their viability assessed with the alamarBlue test. The values of treated cells were normalized to that of untreated controls and reported as mean ± SD.

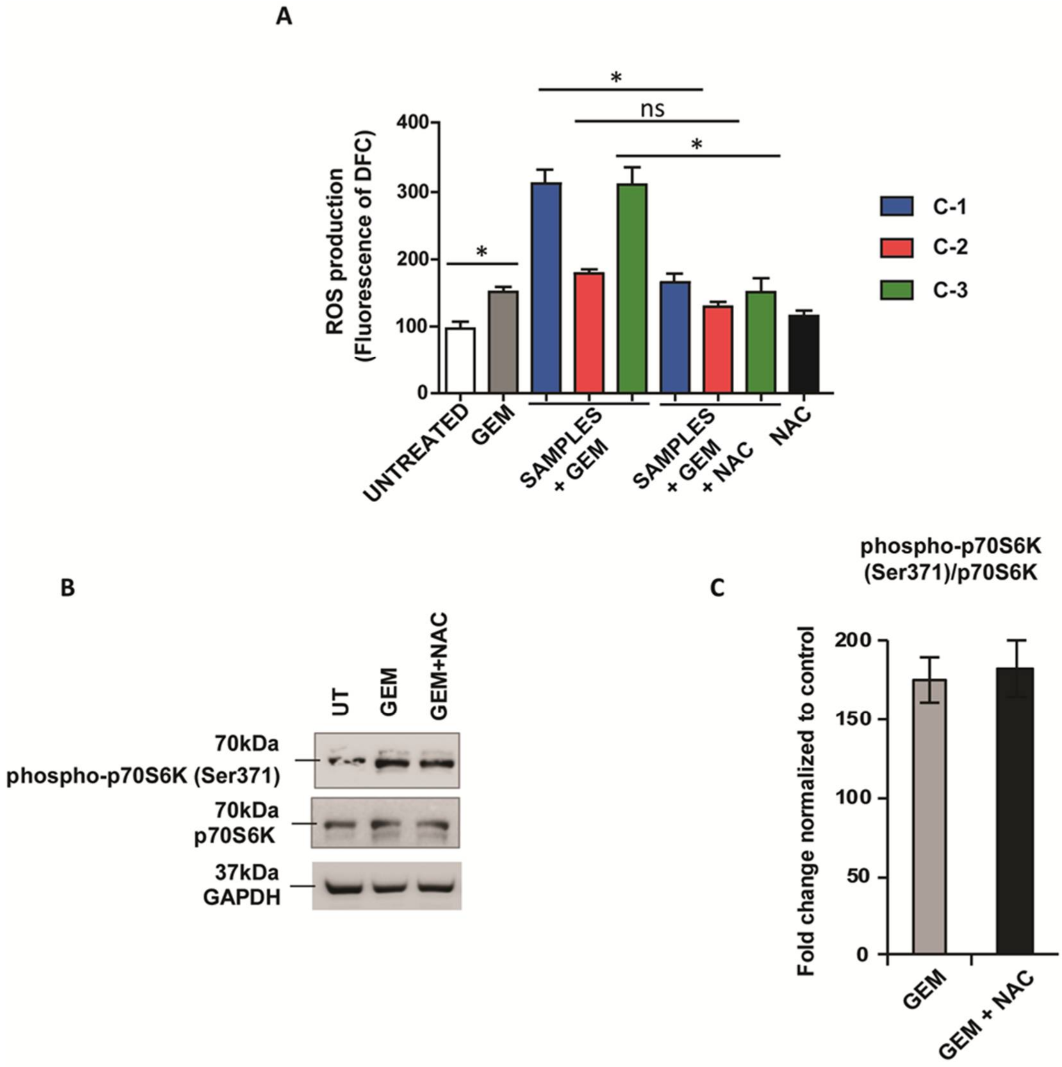

Figure S24. Combined treatment attenuates mTOR pathway in a ROS-dependent manner. A) PANC-1 cancer cell lines treated with 4.5 μM GEM, 500 μM NAC and 1 mM **C-1**, **C-2** and **C-3** for 48 hours. ROS generation was measured as DCF fluorescence, as reported in the methods. The values of treated cells were normalized to that of untreated controls and reported as mean ± SEM. Statistical analysis was performed using one-way ANOVA (each group vs. control)., * $p < 0.05$). B) Western blot analysis of phospho-p70S6K (Ser371) and p70S6K ofPANC-1 cells after treatment with 4.5 μM GEM and 500 μM NAC for 48 hours. GAPDH protein level in the same extract was used as a control loading. C) Densitometry of bands was performed using NIH Image J software and reported as fold change respect to the untreated condition. The values reported are the mean of three experiments and were reported as mean ± SEM.

1 A. L. Upthagrove and W. L. Nelson. Carbinolamines, Imines, and Oxazolidines from Fluorinated Propranolol Analogs. 19F NMR and Mass Spectral Characterization and Evidence for Formation as Intermediates in Cytochrome P450-Catalyzed N-Dealkylation. *Drug Metab. Dispos.*, **2001**, *29*, 1114–1122.
